# Molecular-cytogenetic analysis of *Aegilops triuncialis* and identification of its chromosomes in the background of wheat

**DOI:** 10.1186/s13039-014-0091-6

**Published:** 2014-12-02

**Authors:** Ghader Mirzaghaderi, Andreas Houben, Ekaterina D Badaeva

**Affiliations:** Department of Agronomy and Plant Breeding, Faculty of Agriculture, University of Kurdistan, P. O. Box 66177–15175, Sanandaj, Iran; Leibniz-Institut für Pflanzengenetik und Kulturpflanzenforschung (IPK) Gatersleben, Corrensstraße 3, 06466 Stadt Seeland, Germany; N.I. Vavilov Institute of General Genetics, Russian Academy of Sciences, Moscow, Russia

**Keywords:** *Aegilops triuncialis*, Evolution, Wheat–*Aegilops triuncialis* hybrid, Chromosome marker, Allopolyploidization

## Abstract

**Background:**

Species belonging to the genus *Aegilops* L. are an important source of genetic material for expanding genetic variability of wheat. *Ae. triuncialis* is an allotetraploid in this genus which was originated from hybridization of *Ae. umbellulata* and *Ae. markgrafii* (Greuter) Hammer. Although the *Ae. triuncialis* karyotype was thoroughly examined by conventional chromosome staining and Giemsa C-banding, it is still poorly characterized using FISH markers. The objective of this study was to test the fluorescence *in situ* hybridization (FISH) patterns of *Ae. triuncialis* (2n = 4x = 28, C^t^C^t^U^t^U^t^) chromosomes using different repetitive sequences and to compare the produced patterns to the chromosomes of its diploid ancestors, with the aim of establishing a generalized *Ae. triuncialis* idiogram and detection of *Aegilops* chromosomes in the background of wheat.

**Results:**

The probes pSc119.2-1, pTa535-1, pAs1-1, (CTT)_10_ and the 45S rDNA clone from wheat (pTa71) were hybridized to chromosomes of *Ae. triuncialis* and compared with its diploid progenitors (*Ae. umbellulata* Zhuk., 2n = 2x = 14, UU and *Ae. markgrafii* (Greuter) Hammer, 2n = 2x = 14, CC) and *Ae. cylindrica* Host. (2n = 4x = 28, D^c^D^c^C^c^C^c^), another tetraploid species containing the C-genome. *Ae. cylindrica* was further analyzed by genomic *in situ* hybridization (GISH) using C genome probe in order to identify any possible translocation.

**Conclusions:**

In general, FISH patterns of the U^t^- and C^t^-genome chromosomes of *Ae. triuncialis* were similar to those of U- and C-genome chromosomes of the diploid progenitor species *Ae. umbellulata* and *Ae. markgrafii* respectively, although some differences were observed. Two major 45S rDNA loci were revealed in the short arm of chromosomes A and C, of the C^t^ genome which correspond to homoeologous groups 1 and 5 respectively. Minor 45S rDNA loci were mapped on the short arm of chromosomes 1U^t^ and 5U^t^. GISH analysis revealed three different non-reciprocal homologous or heterologous translocations between C^c^ and D^c^ chromosomes in all studied accessions of *Ae. cylindrica*.

**Electronic supplementary material:**

The online version of this article (doi:10.1186/s13039-014-0091-6) contains supplementary material, which is available to authorized users.

## Background

Species belonging to the genus *Aegilops* L. are an important source of genetic material for expanding genetic variability of cultivated bread wheat, *Triticum aestivum* L. em Thell. (2n = 6x = 42, BBAADD) [1]. The genus *Aegilops* comprises 11 diploid and 12 allopolyploid species [2] with different types of nuclear and cytoplasmic genomes [3]. *Ae. triuncialis* is included in the section *Aegilops* together with diploid *Ae. umbellulata* and several polyploid species sharing the U-genome [2]. *Ae. triuncialis* is subdivided into two subspecies, : ssp. *triuncialis* and ssp. *persica,* which carry the same type of nuclear genome, but different cytoplasmic genomes. *Ae. triuncialis* ssp. *persica* was originated from hybridization of *Ae. umbellulata* as female parent with *Ae. markgrafii* (Greuter) Hammer (syn. *Ae. caudata* L.), whereas ssp. *triuncialis* arose from a reciprocal cross [4,5].

Many accessions of *Ae. triuncialis* are tolerant to biotic and abiotic stresses. It has been exploited for a wide range of traits including resistance to pests and diseases [6-14] and may harbor many other, yet unidentified traits for wheat improvement.

Giemsa C-banding technique has been used to characterize the genomes and chromosomes of wheat and *Aegilops* species [15-17]. In particular C-banding has been employed to examine genetic diversity and to construct the karyotypes of *Ae. umbellulata* [18], *Ae. markgrafii* [19,20], *Ae. triuncialis* [16] and *Ae. cylindrica* [15,21]. Fluorescence *in situ* hybridization (FISH) using repetitive sequences as probes is an alternative powerful technique for chromosome characterization.

Repetitive DNA sequences are major components of the plant genome; in some species they can account for up to 90% of the genome size [22]. Dissimilarity of repetitive DNAs may reflect evolutionary distances between species and these repetitive DNA sequences account for the major differences between genomes [23,24]. The chromosomal localization of various repetitive DNA sequences, including single sequence repeats (SSRs) such as (ACG)_n_ and (GAA)_n_, satellite sequences (pSc119.2, *Afa* family) and ribosomal genes have been used to identify the chromosomes of wheat and its wild relatives [25-28]. Recently, some new tandemly repeated sequences, such as pTa-535, pTa-713, and pTa-86, were also isolated and tested as FISH probes to identify wheat chromosomes [29]. The hybridization patterns of pSc119.2, *Afa* family and rDNA probes were described previously for diploid and polyploid *Aegilops* species [16,30,31]. Some species, like *Ae. umbellulata* [32], *Ae. biuncialis* [33], *Ae. cylindrica* [21] were studied in more detail. These studies showed that combinations of pSc119.2 and *Afa* probes in most species do not permit the complete identification of all chromosomes, because pSc119.2 probe hybridized mainly to subtelomeric chromosome regions, while the *Afa* family produces just few signals on chromosomes of the S-genome group, T-, U- and C-genomic species. To solve this problem some authors [26,34,35] suggested to use two or three base-pair synthetic oligo probes as diagnostic markers. These studies demonstrated that the GAA microsatellite is valuable to identify chromosomes of the wheat A- and B-genomes. The labeling patterns generated with this sequence in general corresponded to the Giemsa N-banding patterns of the respective chromosomes thus allowing linking the FISH and Giemsa N- or C-banding analyses of wheat. The GAA repeat was further used to characterize the chromosomes of some *Aegilops* species including *Ae. biuncialis, Ae. comosa* and *Ae. umbellulata* [33].

The *Ae. triuncialis* karyotype was thoroughly examined by conventional chromosome staining [36] and C-banding [16], however it is still poorly characterized using FISH markers. Although Badaeva et al. described the labeling patterns of pSc119.2, pTa71, pTa794 DNA probes on *Ae. triuncialis* chromosomes, their correlation with a pattern of Giemsa staining was established only on the basis of chromosome morphology [16]. Morphological similarity of many *Ae. triuncialis* chromosomes however can impede their correct classification. Hence the main objective of this study was to analyze the FISH patterns of *Ae. triuncialis* chromosomes using different repetitive sequences and to compare the produced patterns to the chromosomes of its diploid ancestors, with the aim of establishing a generalized *Ae. triuncialis* idiogram for facilitating the accurate chromosome identification, tracing possible chromosome changes over the course of evolution and detection of *Ae. triuncialis* chromatin introgressed into wheat. Another tetraploid *Aegilops* species carrying the C-genome – *Ae. cylindrica* – was taken for the comparison of the C-genome chromosomes in a different genetic background.

## Results and discussion

To develop an informative combination of FISH markers that allows precise identification of all *Ae. triuncialis* chromosomes, the following combinations of probes were tested: pSc119.2-1 + pTa535-1, pSc119.2-1 + pAs1-1, pSc119.2-1 + (CTT)_10_ and pTa71.

**Figure 1:**
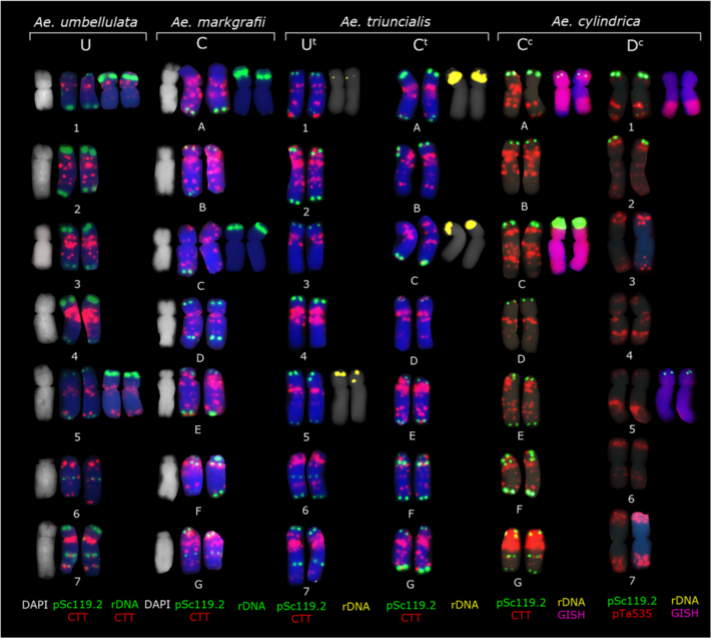
**Representative karyotypes of** ***Ae. umbellulata*** **(accession S234),** ***Ae. markgrafii*** **(accession AE1418),** ***Ae. triuncialis*** **(accession S197) and** ***Ae. cylindrica*** **after FISH with different repetitive DNA probes.** The yellow signals on *Ae. triuncialis* chromosomes represent 45S rDNA with the sizes relevant to the intensity of produced signals. The probe combination on each genome chromosomes is presented at the bottom of the figure with the related colour.

Examination of two accessions each of diploid *Ae. umbellulata* and *Ae. markgrafii* showed only few within-species hybridization pattern polymorphisms, though some minor differences were observed between *Ae. markgrafii* accessions. All *Ae. markgrafii, Ae. umbellulata* and *Ae. triuncialis* accessions analyzed showed similar hybridization patterns with the probe pSc119.2-1, which hybridized predominantly to the subtelomeric regions of one or both arms of all chromosomes. Additional interstitial signals were observed on the long arm of chromosomes 6 and 7 of the U-genome and chromosomes F and G of the C-genome (Figure 1). Thus, these chromosomes can be distinguished using pSc119.2-1 alone. However, the labeling pattern of *Ae. triuncialis* chromosomes should be treated with care because few minor changes in the position and signal intensity have been recorded for some chromosomes. The most distinct changes were observed for chromosome G, which lost a marker pSc119.2 site in the middle of the long arm, but acquired an increased site in the telomeric region of the same arm (Figure 1).

Our result confirms previous observations that probe pAs1 is not informative for the identification of U- and C-genome chromosomes [16,30,33], because it generates only few weak signals on only some chromosomes of these genomes. *In situ* hybridization using the (CTT)_10_ repeat allowed the identification of all seven chromosomes of *Ae. markgrafii* and *Ae. umbellulata* (Figure 1). (CTT)_10_ hybridization patterns on *Ae. triuncialis* chromosomes were comparable to those on the ancestral species, however some minor differences were observed in signal distribution and intensity (Figure 1). In particular, we revealed a significantly reduced number of hybridization sites in the long arm of chromosome A, which possessed profound signals in pericentromeric regions of short and long arms and two weak telomeric sites. Another NOR-bearing chromosome – C, carried large signals in proximal region of both arms and a small site in subtelomeric region of the long arm, whereas in the parental species we observed numerous, relatively weak interstitial signals of the same probe distribute over the length of the long arm. Labeling of pericentromeric region of chromosome E was much stronger in *Ae. triuncialis* than in *Ae. caudata* karyotype. By contrast, pericentromeric signals on chromosome F were smaller in allotetraploid species as compared to the diploid progenitor.

The (CTT)_10_ hybridization pattern of *Ae. markgrafii* and *Ae. umbellulata* chromosomes was similar with previously reported pattern on *Ae. umbellulata* chromosomes [33] and basically coincided with the position of previously reported Giemsa C-bands [18,19]. Similarity of the hybridization patterns on *Ae. triuncialis* chromosomes compared to *Ae. markgrafii* and *Ae. umbellulata* indicates that the C- and U-genomes have not underwent the significant structural changes relative to the parental species.

FISH analysis using labeled 45S rDNA showed four pairs of *Ae. triuncialis* chromosomes with rDNA signals of different size (Figure 1). A suppression of nucleolar organizing regions of the C-genome chromosomes in *Ae. triuncialis* was suggested earlier by [37] based on Ag-NOR staining and then by [16] based on FISH with two rRNA gene families. Our results also confirm suppression of 45S rRNA gene loci on one of the parental genome of *Ae. triuncialis*, however, with the use of genomic *in situ* hybridization (GISH) followed by FISH with pTa71 probe we showed that two major loci are located in the short arm of the C-genome chromosomes A and C, but not on the chromosomes A (1C^t^) and 5U^t^, as was suggested in [16]. The discrepancies in classification of NOR-bearing chromosome 5 can be due to the fact that in a previous paper the genome affinity of chromosomes was determined by their morphology and therefore should be considered as tentative. Two minor NOR clusters are located in the short arms of chromosomes 1U^t^ and 5U^t^. The signal size decreased in the order A(1C^t^) > C(5C^t^) > 5U^t^ > 1U^t^ (Figure 1). Differences in signal size on chromosomes 1U^t^ and 5U^t^ observed in our study compared to the previous one can be due to intraspecific polymorphism as we used different accessions of *Ae. triuncialis.*

Each of the diploid species *Ae. markgrafii* and *Ae. umbellulata* carries two pairs of NOR-bearing chromosomes that were identified as chromosomes A = 1C and C = 5C in *Ae. markgrafii* and 1U and 5U in *Ae. umbellulata*, respectively. FISH analysis suggests that in *Ae. triuncialis* elimination of most of the rRNA gene copies occurred from the U^t^ genome of *Ae. umbellulata* (Figure 1). A similar loss of 45S rRNA genes occurred in the A/A^t^ genome of *Triticum turgidum* and *T. timopheevii* [38] after allopolyploidization.

**Figure 2:**
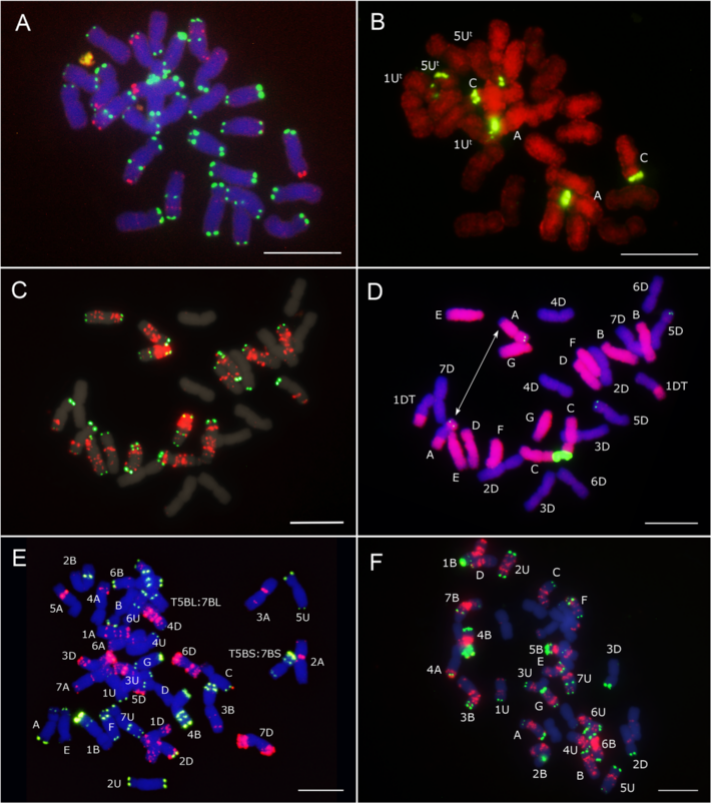
**FISH and GISH patterns of** ***Ae. triuncialis*** **,** ***Ae. cylindrica*** **and F**_**1**_ **hybrids derived from wheat and** ***Ae. triuncialis*** **crosses. A**: FISH signals from oligonucleotide probes pAS1-1 (FAM 5′ labeled), pSc119.2-1 (Tamra labelled) on the *Ae. triuncialis* (accession S197); **B**: Re-probing on the same preparation using *Ae. markgrafii* genomic DNA (red signals) and pTa71 clone (green signals); **C**: FISH signals from oligonucleotide probes pSc119.2-1 (FAM 5′ labeled), pTa535-1 (Tamra labelled) and (CTT)_10_ (Cy3 labelled) on the *Ae. cylindrica* ecotype. Just signals of (CTT)_10_ (mainly on C^c^ chromosomes) and pSc119.2-1 are presented in this picture. Using Texas Red filter, pTa535-1 signal was observed and identified D^c^ chromosomes which are presented in Figure 1; **D**: Re-probing on the same preparation using *Ae. markgrafii* genomic DNA (red signals) and pTa71 clone (green signals) which shows unbalanced translocation on chromosome pair 1D and 3C. The translocation on 3C is heterologous; **E**: FISH pattern of repetitive oligonucleotide probes pSc119.2-1 (FAM 5′ labelled), pTa535-1 (Tamra labelled) on mitotic metaphase chromosomes of a derived F_1_ hybrid *T. aestivum* cv ‘Zarin’- *Ae. triuncialis* (accession S101); **F**: FISH pattern of repetitive oligonucleotide probes pSc119.2-1 (FAM 5′ labelled) and (CTT)_10_ (Cy3 labelled) on mitotic metaphase chromosomes of a derived F_1_ hybrid *T. aestivum* cv ‘Pishgam’- *Ae. triuncialis* (accession S101). Bar = 10 μm.

A mitotic metaphase cell of the F_1_ hybrid *T. aestivum* cv ‘Zarin’-*Ae. triuncialis* (n = 5x = 35, genomically BADU^t^C^t^) after FISH using pSc119.2-1 and pTa535-1 as probe combination is shown in Figure 2E. All A-, B- and D-genome chromosomes of wheat can be identified by their characteristic repetitive sequences patterns, arm ratios and chromosome sizes. A 5B:7B translocation was identified in the background ‘Zarin’ (Figure 2E). All U^t^- and C^t^-genome chromosomes of *Ae. triuncialis* can be identified, however a combination of pSc119.2-1 and (CTT)_10_ probes proves to be most useful for the identification of all individual *Ae. triuncialis* chromosomes in wheat background (Figure 2F) because pTa535-1 probe does produce only few signals on the C^t^ or U^t^ chromosomes.

So far, the homoeologous relationship of only three chromosomes of *Ae. markgrafii* has been established. Analysis of the chromosome substitution lines A (1C), C (5C) [19] and B (2C) [39] indicated that the chromosomes A, B and C should be assigned to homoeologous groups 1, 2 and 5 of wheat, respectively. The *Ae. markgrafii* chromosomes - D, E, F, and G showed homoeology to more than one group, based on isozyme [40] and restriction fragment length polymorphisms (RFLP) [20,41] analyses. Structural rearrangements of the U-genome chromosomes of *Ae. umbellulata* have been earlier deduced based on comparative chromosome mapping [42]. That is why both *Ae. markgrafii* and *Ae. umbellulata* have highly asymmetrical karyotypes, compared to the more symmetrical karyotypes of the other diploid species of this genus [36].

The C-genome is also present in another tetraploid *Aegilops* species with a different genome constitution, *Ae. cylindrica* (D^c^D^c^C^c^C^c^) [43]. *Ae. triuncialis* and *Ae. cylindrica* is suggested to evolve rather recently with only few modifications of the parental genomes [44]. To find possible changes of the C-genome due to polyploidization, we included *Ae. cylindrica* into the analysis of the C-genome evolution. The pattern produced by the (CTT)_10_ repeat on C^c^-genome chromosomes of *Ae. cylindrica* was more similar to that in the ancestral species *Ae. markgrafii* than the C^t^-genome chromosomes of *Ae. triuncialis*. However, three different non-reciprocal homologous or heterologous translocations between C^c^ and D^c^- genome chromosomes where observed in all the studied accessions of this species. Thus, *Ae. cylindrica* accession S376 carried an intergenomic translocation (Additional file 1: Figure S1) and the *Ae. cylindrica* ecotype consisted of genotypes with two different translocations (Figure 2D and Additional file 1: Figure S1). Reciprocal translocations between C^c^ and D^c^ chromosomes have been reported in this species previously [21]. Sequential FISH and GISH showed that the breakpoints were mainly located near the (CTT)_10_ hybridization sites. Similar relationship between intergenomic translocation breakpoints and SSR-rich chromosomal regions in the allopolyploid species has been reported by other authors [33] which suggests that SSR DNA sequences might facilitate the formation of chromosomal rearrangements. Frequent incidence of reciprocal and non-reciprocal translocations can be an indicative of an extensive speciation process in this relatively new allopolyploid species. A more recent origin of *Ae. cylindrica* than *Ae. triuncialis* can also be presumed based on the higher similarity of the C^c^-genome chromosomes with the C-genome chromosomes of diploid *Ae. markgrafii* as compared to the C^t^-genome of *Ae. triuncialis*. We could hardly discriminate the C^t^ and U^t^ genome chromosomes of *Ae. triuncialis* (accession S197) by GISH using *Ae. markgrafii* as probe (Figure 2A and B). This can be due to very close evolutionary relationships of these genomes which share many repetitive sequences [44] thus precluding their discrimination. No intergenomic translocations was detected in the accessions of *Ae. triuncialis* used.

## Conclusion

In conclusion, FISH patterns of the U^t^- and C^t^-genome chromosomes of *Ae. triuncialis* using different repetitive were similar to those of U- and C-genome chromosomes of the diploid progenitor species *Ae. umbellulata* and *Ae. markgrafii* respectively, although some differences were observed. *In situ* hybridization using the (CTT)_10_ repeat allowed the identification of all chromosomes of *Ae. triuncialis* and its diploid ancestors *Ae. markgrafii* and *Ae. umbellulata. Ae. triuncialis* chromosomes could also be identified in the background of bread wheat. GISH analysis revealed different non-reciprocal homologous or heterologous translocations between C^c^ and D^c^ chromosomes in the studied accessions of *Ae. cylindrica*.

## Methods

### Plant materials

Two accessions of *Ae. markgrafii* (AE1418, AE1082), two accessions of *Ae. umbellulata* (S147, S234), three accessions of *Ae. triuncialis* (S101, S146, S197), and one accession (S376) and one ecotype (collected in the Kurdistan province, Iran) of *Ae. cylindrica*, were examined. The *Ae. markgrafii* accessions were obtained from the germplasm collections of the IPK, Germany and other accessions are maintained in the Research Institute of Forests and Rangelands (RIFR) of Iran. *T. aestivum* cultivars ‘Zarin’ and ‘Pishgam’ were crossed with *Ae. triuncialis* (accession S101) and the resulted F_1_ seeds were used for identification of *Ae. triuncialis* chromosomes in the background of wheat.

### DNA probes

The 5′ with 6-carboxyfluorescein (6-FAM) or 6-carboxytetramethylrhodamine (Tamra) end-labelled oligo probes oligo-pAs1-1 (Tamra-5′-CCT TTC TGA CTT CAT TTG TTA TTT TTC ATG CAT TTA CTA ATT ATT TTG AGC TAT AAG AC-3′), oligo-pSc119.2-1 (6-FAM-5′-CCG TTT TGT GGA CTA TTA CTC ACC GCT TTG GGG TCC CAT AGC TAT-3′) and oligo-pTa535-1 (Tamra-5′-AAA AAC TTG ACG CAC GTC ACG TAC AAA TTG GAC AAA CTC TTT CGG AGT ATC AGG GTT TC-3′) [29,45] were synthesized by MWG (Germany). The oligo (CTT)_10_ probe was directly labelled with 5/6 sulforhodamine 101PEG3_azide by click chemistry (Baseclick). The plasmid pTA71 containing the 45S rDNA repeat from wheat was directly labelled by nick translation and used to detect the NORs.

### FISH and GISH

Root tips were pretreated with ice cold water for 24 h and were then fixed in ethanol-glacial acetic acid (3:1). Mitotic chromosome spreads were prepared using dropping technique. FISH was carried out according to [33]. After documentation of the FISH signals, some of the preparations were rehybridized to detect the 45S rDNA sites or the parental genomes by genomic *in situ* hybridization (GISH).

GISH was carried out after FISH on the same preparations. Therefore, total *Ae. markgrafii* genomic DNA was labelled with Atto-550 11-dUTP by nick translation, and used as a probe to detect the C-genome chromosomes of *Ae. triuncialis* and *Ae. cylindrica*. Unlabelled, fragmented wheat DNA was used as blocking DNA at 60 times the quantity.

## Additional file

Additional file 1: Figure S1 A GISH using labelled *Ae. markgrafii* genomic DNA on a mitotic metaphase cell of the *Ae. cylindrica* ecotype showing unbalanced intergenomic translocations on two pairs of chromosomes. The red labelling is the GISH with the C genome, and the green is the rDNA loci. This ecotype consisted of genotypes with two different translocations. The other one is shown in Figure 2D; B: *Ae. cylindrica* accession S376 showed an unbalanced intergenomic translocation on one pair of chromosomes. Bar = 10 μm.
