# Supplementary material for: Molecular-cytogenetic analysis of Aegilops triuncialis and identification of its chromosomes in the background of wheat
Source: Mol Cytogenet. 2014 Dec 2;7:91. doi: 10.1186/s13039-014-0091-6 (PMC4263106; doi:10.1186/s13039-014-0091-6)
Supplement: Additional file 1: Figure S1 — A GISH using labelled Ae. markgrafii genomic DNA on a mitotic metaphase cell of the Ae. cylindrica ecotype showing unbalanced intergenomic translocations on two pairs of chromosomes. The red labelling is the GISH with the C genome, and the green is the rDNA loci. This ecotype consisted of genotypes with two different translocations. The other one is shown in Figure 2D; B: Ae. cylindrica accession S376 showed an unbalanced intergenomic translocation on one pair of chromosomes. Bar = 10 μm. [file 13039_2014_91_MOESM1_ESM.docx]

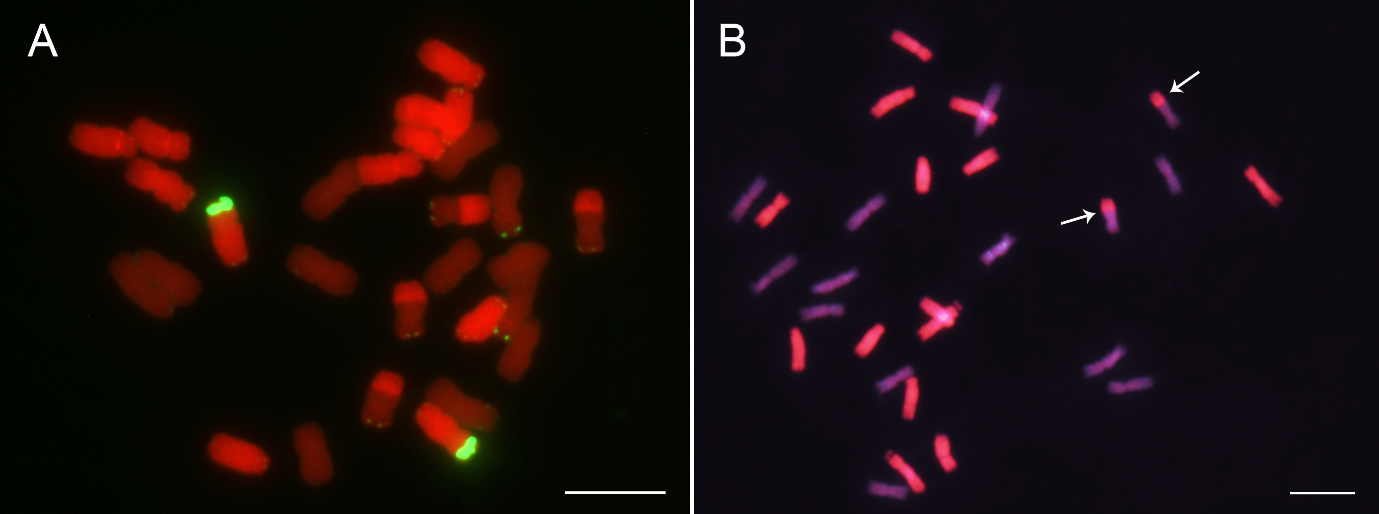


**Figure S1 A**: GISH using labelled *Ae. markgrafii* genomic DNA on a mitotic metaphase cell of the *Ae. cylindrica* ecotype showing unbalanced intergenomic translocations on two pairs of chromosomes. The red labelling is the GISH with the C genome, and the green is the rDNA loci. This ecotype consisted of genotypes with two different translocations. The other one is shown in figure 2D; **B**: *Ae. cylindrica* accession S376 showed an unbalanced intergenomic translocation on one pair of chromosomes**.** Bar = 10 μm.
